# School Bullies’ Intention to Change Behavior Following Teacher Interventions: Effects of Empathy Arousal, Condemning of Bullying, and Blaming of the Perpetrator

**DOI:** 10.1007/s11121-016-0712-x

**Published:** 2016-10-01

**Authors:** Claire F. Garandeau, Annina Vartio, Elisa Poskiparta, Christina Salmivalli

**Affiliations:** 1 Department of Pedagogical and Educational Sciences, Utrecht University, Heidelberglaan 1, 3584 CS Utrecht, The Netherlands; 2 Åbo Akademi, Turku, Finland; 3 University of Turku, Turku, Finland

**Keywords:** Bullying, Intervention, School, Empathy, Anti-bullying program

## Abstract

This study examines how bullies’ perceptions of how they were treated by a teacher (or other school personnel) during discussions aimed at putting an end to bullying influenced their intention to change their behavior. After each discussion, which took place as part of the implementation of an anti-bullying program, bullies anonymously reported the extent to which they felt that the teacher aroused their empathy for the victim, condemned their behavior, or blamed them. Half of the schools implementing the program were instructed to handle these discussions in a confrontational way—telling the bully that his behavior is not tolerated—while the other half were instructed to use a non-confronting approach. Schools were randomly assigned to one of the two approaches. A total of 341 cases (188 in primary and 153 in secondary schools) handled in 28 Finnish schools were analyzed. Regression analyses showed that attempts at making bullies feel empathy for the victim and condemning their behavior both increased bullies’ intention to stop. Blaming the bully had no significant effect. Bullies’ intention to change was the lowest when both empathy-arousal and condemning behavior were low. The effects of empathy arousal were stronger when condemning the behavior was low (and vice versa), suggesting that teachers tackling bullying should make sure to use at least one of these strategies. When choosing not to raise the child’s empathy, clear reprobation of the behavior is key.

The dramatic and enduring consequences of school bullying, an intentional abuse of power against peers, have been broadly documented: Being exposed to bullying by peers can lead to increases in a wide range of mental health problems (Reijntjes et al. 2010) and engaging in school bullying predicts higher rates of violence later in life (Ttofi et al. 2012). These adverse outcomes leave little doubt about the necessity to reduce such behavior. The last decades have seen a marked increase in the development of anti-bullying interventions, encouraging teachers and other school personnel to take an active role in countering the bullying incidents coming to their attention. Most meta-analyses on the effectiveness of anti-bullying programs concur that, overall, they lead to decreases in bullying behaviors (e.g., Baldry and Farrington 2007; Evans et al. 2014; Ferguson et al. 2007; Ttofi and Farrington 2011, 2012). Higher reductions are obtained when programs are more intensive and include meetings with parents, firm disciplinary methods, and higher playground supervision (Ttofi and Farrington 2011). However, the effectiveness of these programs remains limited—on average, reductions in bullying and victimization being around 20 %—and a better knowledge of the mechanisms underlying these positive effects is strongly needed (Bradshaw 2015).

With regard to interventions targeted at bullies in particular, two major approaches can be distinguished: The first is a disciplinary, confronting approach, which involves condemning the bullying behavior and holding the bully responsible for what happened (Olweus 1993). This approach is often referred to as the traditional approach to handling cases of bullying, as it is the most commonly used (Burger et al. 2015) and was the primary approach advocated for anti-bullying intervention in the 1970s and 1980s. The second is a non-confronting approach, which emerged in the 1990s with the release and implementation of the shared concern method (Pikas 1989) and the no-blame approach (Robinson and Maines 1997). The key elements of this non-confronting approach are the arousal of bullies’ empathy for their victims (i.e., the ability to feel or imagine the victim’s emotions) and the absence of accusations toward bullying perpetrators. This approach assumes that empathic concern cannot be elicited if the bullies feel blamed (Rigby 2007; Robinson and Maines 1997, 2008).

Each approach has its proponents and its critics, and the controversy over which one should be recommended to school personnel still lingers (Smith 2014) for several reasons: First, there is no compelling evidence that one of these approaches is more effective than the other (Garandeau et al. 2014b). Second, research on the effectiveness of such approaches has generally relied on adults’ reports of the approach that they used instead of bullies’ reports of how they experienced the intervention. More so than the intended approach itself, young bullies’ perceptions of how they were treated by the adult should determine whether they will cease or pursue their conduct. Third, it is likely that, in practice, teachers combine elements of different approaches, such as blaming the bully for his behavior *and* trying to increase his empathy for the victim. The effects of these specific elements may add up or interact, but this cannot be determined by simply comparing the effects of the two main approaches; it requires an examination of the main and interactive effects of these specific components. Finally, there might be important variability in implementation of the confronting approach (see Ayers et al. 2012). Its effectiveness may depend on whether the emphasis is on condemning the behavior or blaming the child himself (see Braithwaite 1989). Little is known, however, on the differential effects of these two forms of disciplinary actions on bullying behavior.

The present study aims to fill these gaps in the literature, by examining the unique and interactive effects of teachers’ use of empathy arousal, condemning of bullying behavior and blaming of the perpetrator on bullies’ intention to change behavior. Using a sample of children and adolescents who engaged in bullying and were exposed to discussions by school personnel, we examined bullies’ perceptions of the extent to which the adult used each of these three strategies rather than adults’ reports of the strategies they employed. First, we tested for the main effects of perceived empathy arousal, condemning of bullying, and blaming of the perpetrator on bullies’ intention to stop. Second, we tested whether the effects of perceived empathy arousal depended on the extent to which bullies perceived that the adult blamed them and/or the extent to which they perceived the adult condemned their behavior.

## Adult Interventions in Cases of Bullying in Schools

Teachers and other adults in schools have a key role to play in the prevention and cessation of bullying: Bullying episodes are more frequent and possibly more severe when adult supervision is lacking (Craig and Pepler 1997) or when teachers ignore the incident (Yoon 2004). Teachers’ efforts at reducing bullying, as perceived by their students, have also been found to be associated with a decrease in bullying over time (Veenstra et al. 2014). When teachers become aware of bullying incidents, they generally choose to take action rather than ignore the incident (Bauman et al. 2008; Burger et al. 2015). However, these teacher interventions are successful at making the bullying stop only about half of the time and can aggravate the situation in some cases (e.g., Fekkes et al. 2005; Nixon and Davis 2011). Therefore, more research is needed to determine what makes teachers’ interventions effective or not.

When presented with a hypothetical bullying scenario and a questionnaire of possible responses, school personnel are more likely to endorse authority-based interventions, such as disciplining the bully through verbal reprimands or more severe sanctions, over non-confrontational strategies, such as discussing with the bully ways to improve the situation or sharing their concern about the victim with the bully (Bauman et al. 2008; Burger et al. 2015). In these studies, victim-focused interventions, such as telling the victim to stand up to the bully were the least likely to be chosen. Among bully-focused interventions, the debate has centered around the confronting, disciplinary approach and the non-confronting, non-blaming approach. Surprisingly, these two main approaches have never been directly compared, with the exception of the study by Garandeau et al. (2014b). The relative effectiveness of each approach was measured by asking victims, 2 weeks after the intervention, whether the bullying had stopped. None of the approaches was shown to be significantly more effective than the other overall, after controlling for school level, type of aggression, and duration of victimization. Rather, the effectiveness of each approach depended on other factors: The confronting approach was better than the non-confronting approach in secondary school but not in primary school, and in cases of short-term victimization but not in cases of long-term victimization. While this study examined the link between the approach school personnel were instructed to use and the cessation of bullying according to the victim, it did not consider the perspective of the bully (i.e., his perception of the intervention and his decision to stop). A better understanding of what makes anti-bullying interventions effective requires a careful examination of bullies’ cognitions in response to these interventions.

The focus on these main approaches has prevented us from looking at the effectiveness of their specific components, such as raising the bully’s empathy for the victim. One of the key assumptions of the non-confronting approach is that trying to raise bullies’ empathy should be less effective if bullies are openly reprimanded (Rigby 2007; Robinson and Maines 1997, 2008). However, empirical evidence showing whether this is actually the case is still lacking. Furthermore, it is possible in practice to combine elements of both approaches, such as blaming the perpetrator and attempting to raise his empathic concern for the victim. Therefore, there is an urgent need to examine the effects of these individual components separately in order to determine which combination yields the best results.

## Does Raising Empathy Work?

Empathy is generally defined as the ability to feel or imagine another person’s emotions and has both affective and cognitive components. Cognitive empathy refers to the ability to understand the emotions of another person, while affective empathy refers to the ability to experience how another feels (Cohen and Strayer 1996). These capacities are thought to deter individuals from aggressing against others. Children and adolescents who engage in bullying have consistently been found to have lower empathy, in particular affective empathy (Caravita et al. 2009; Jolliffe and Farrington 2011; van Noorden et al. 2015). Longitudinal investigations suggest that low empathy may lead to increases in bullying over time (Stavrinides et al. 2010). Empirical support for the link between bullying behavior and empathic deficiencies explains why many anti-bullying interventions aim at increasing the empathy of students who bully their peers. This is typically done by pointing out to the bullies how difficult the situation is for the victim. Until recently, there was little evidence that the empathy levels of young bullies could be raised and in turn decrease their bullying behavior.

Recent experimental studies suggest that attempts at raising the empathy of young aggressors may be successful at altering their behavior. In an experiment by van Baardewijk et al. (2009), children played a computer-based competitive game against a simulated opponent, and aggression was assessed by the intensity of the noise that they chose to blast at their opponents. Children higher in psychopathic traits were more aggressive, except when the distress of the target was made salient through a written message expressing his or her fear. In another experiment, using a sample of sixth graders identified as bullies (Sahin 2012), participation in an empathy-training program was found to increase empathic skills and decrease bullying. Attempts at raising school bullies’ empathy by emphasizing their victims’ suffering may therefore be effective at reducing their behavior.

## Does Blaming Work?

Blaming bullies consists of holding them clearly responsible for the bullying and can involve taking disciplinary actions, such as contacting the bully’s parents or the principal. Whether this is an effective way to reduce bullying has been highly controversial. One of the reasons why no-blame approaches were developed was to avoid bullies’ refusal to cooperate as well as possible retaliation against the victim that may result from adults’ accusations of bullies (Rigby 2007; Robinson and Maines 1997). In other words, blaming bullies was believed to make discussions counter-productive. Cross-sectional studies on the effects of parenting styles on children’s behaviors support the idea that blaming bullies may not be the optimal strategy: In a large sample of fourth to seventh graders, Ahmed and Braithwaite (2004) found that parents of bullies were more likely than parents of non-bullies to endorse an authoritarian parenting style, operationalized as guilt induction (“I let my child know how ashamed and disappointed I am when he/she misbehaves”) along with punishment, control, and negative affect. Using the same sample, Ahmed (2001) examined parental use of stigmatizing shaming by presenting parents with hypothetical school bullying scenarios in which their own child misbehaved; parents who endorsed statements indicating that their child meant to do what he did and would repeat this behavior in the future were more likely to have children reporting to initiate bullying against other children. On the other hand, meta-analyses of whole anti-bullying programs have found that the use of disciplinary methods, such as serious talks with bullies, sending them to the principal, or loss of privileges—which are forms of punishment and imply that bullies are blamed for the behavior—was associated with better results (Ttofi and Farrington 2011).

One plausible explanation for these seemingly contradictory findings is that no distinction was made between condemning the behavior and blaming the perpetrator. It may be a key distinction to make as the effectiveness of authority-oriented approaches may rely on condemning the behavior only. Expressing disapproval of the behavior sets clear limits about what is acceptable and what is not. As a behavior can be changed, focusing on the behavior emphasizes the possibility for change. It is a judgment of an event as bad or wrong and differs from blaming the child himself, which is potentially damaging (see Malle et al. 2014). Blaming or devaluing the person conveys the message that there is something wrong with what he is as an individual. Such accusations may both increase anger and imply that bullying is a stable characteristic of the child, which in turn may encourage the child to continue bullying. In the current study, we were able to distinguish between bullies’ perceptions of the adult’s blaming of themselves and bullies’ perceptions of the adult’s condemning of their behavior.

## The Present Study

This study investigates young bullies’ perceptions of how they were treated by school adults during discussions designed to put an end to their bullying behaviors. After the discussion, they anonymously filled out a questionnaire that assessed the extent to which they felt that the teacher aroused their empathy for the victim, condemned their behavior, blamed them, as well as their feelings of self-efficacy for behavioral change. Variation in the adults’ handling of the discussion was introduced by instructing half of them to use a confronting approach and the others to use a non-confronting approach. Our main objective was to examine whether and how perceived empathy arousal, condemning of the behavior and blaming of the perpetrator would be associated with bullies’ reported intention to change their behavior, controlling for school level (primary vs secondary), type of approach, and feelings of self-efficacy for change. We hypothesized that both perceived empathy arousal and condemning of the behavior would be positively associated with intention to change. We expected, however, that feeling blamed by the adult would not. Our second objective was to examine whether the effects of perceived empathy arousal would depend on bullies’ perception of the adult’s condemning of their behavior and on the adult’s blaming of themselves. We hypothesized that the effects of perceived empathy arousal would be higher when condemning of the behavior was higher but not when blaming of the bully was higher.

## Method

### Sample

**Fig. 1:**
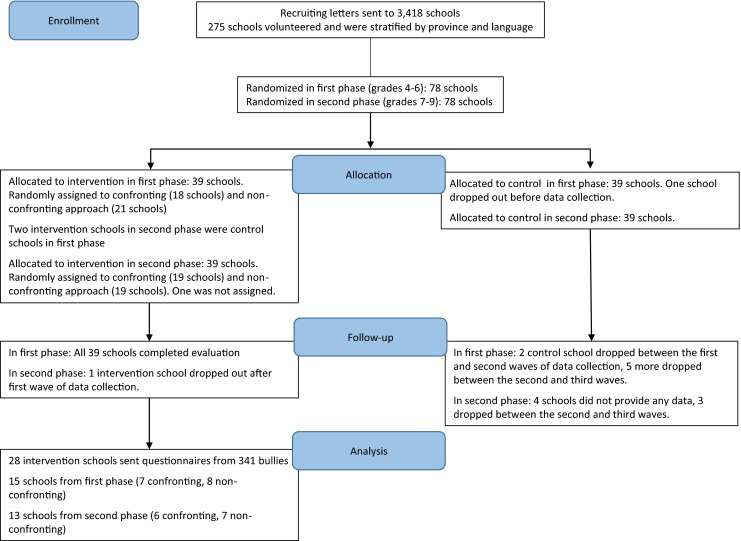
Flowchart of the recruitment and allocation of schools for grades 4–6 and grades 7–9 of the KiVa program pilot evaluation

The current study was conducted within the implementation of a whole-school anti-bullying intervention, the KiVa program. This program was developed in Finland and its effectiveness was evaluated by a randomized controlled trial (RCT) in 2007–2008 for grades 4–6 and in 2008–2009 for grades 1–3 (not considered in the present study) and 7–9 in 78 control schools and 78 intervention schools (including 39 primary schools and 39 secondary schools; see Fig. 1). The schools were randomly selected through a procedure described in detail elsewhere (Kärnä et al. 2011). The program includes both universal and indicated actions. Universal actions are meant for all students and include, among other things, a series of student lessons on topics such as the importance of respect in relationships, group pressure, and bullying. Indicated actions, on the other hand, are meant only for victims and bullies and are the focus of the present study. Every time a case of bullying was witnessed or revealed, schools were instructed to organize separate discussions with victims and bullies (see description of the full procedure below). Through random assignment, half of the intervention schools were instructed to use a confronting approach and the other half a non-confronting approach for the discussions with the bullies. The non-confronting approach was used by 40 schools (21 primary and 19 secondary) and the confronting approach by 37 schools (18 primary, 19 secondary). The effects of these two approaches on the perpetuation of bullying are compared in the study by Garandeau and colleagues (2014b), who analyzed victims’ reports about 2 weeks after the intervention took place. The data analyzed in the present study are derived from questionnaires anonymously filled out by bullies in intervention schools, during the RCT phase. They filled them out immediately after their first discussion with the teacher (or other school staff member). The questionnaires were administered only to bullies with a signed parental permission.

Among the 78 intervention schools, only 28 (including 15 primary and 13 secondary schools) sent a total of 341 questionnaires back to the research team. The number of cases per school ranged from 2 to 33, due to variation in the number of bullying cases handled in the intervention schools during the RCT. Among the 341 cases analyzed, 133 had been handled with the confronting approach (63 in primary schools, 70 in secondary schools) and 208 with the non-confronting approach (125 in primary schools, 83 in secondary schools). Due to the anonymity of the questionnaire, no further information was available on the participants.

### Procedure

#### Indicated Actions

In each school implementing the KiVa program, an anti-bullying team called the *KiVa team* was formed. The teams typically consisted of three adults from the school personnel. Every time a case of bullying came to their attention, they launched a five-step process: (1) a screening to determine whether this was actually bullying, and not a mere conflict between students, (2) a discussion with the victim, (3) a discussion with the bully(ies), (4) a follow-up discussion with the victim, and (5) a follow-up discussion with the bully(ies). In addition, the classroom teacher was requested to encourage a few prosocial classmates to support the victimized peer. The members of the KiVa team were instructed to hold the first discussions with the bullying students—which are the focus of the present study—as soon as possible after the case had come to their attention. As recommended by Pikas (2002) for this kind of intervention, these discussions were planned so as to come as a surprise to the bullies; this prevents them from discussing the matter with their peers before meeting with the KiVa team. The adults were asked to keep these discussions short and focused and to keep a written record of the agreements made during the discussions.

#### The Two Approaches

The discussions with the bullies were conducted using either a confronting approach or a non-confronting approach. The KiVa team members received training for the specific approach that their school had been prescribed to use, as well as supervision three times a year in the form of group meetings, where members of three different KiVa teams met to evaluate their work together with a supervisor from the research team. The two approaches have several similarities: The discussion starts with a presentation of the issue, which is that a particular child has been bullied; it ends with a request to the bully with regard to his/her future behavior (e.g., “What are you going to do now?”). A commitment to change behavior is expected from the bully. However, the two approaches differ significantly in several respects. In discussions using the confronting approach, the adults had to make it clear to the student that they knew about the bullying, that bullying was not accepted in the school, and that the bullying had to cease immediately. The tone of the discussion was to be condemning rather than understanding. With this approach, the perpetrator is openly held responsible for what happened. The goal of the non-confronting approach, on the other hand, was to arouse the bully’s concern for the victim—or create a *shared concern* (Pikas 2002). The focus was on reaching an agreement that the bullied student must be feeling bad considering the negative things that he or she is experiencing at school. Furthermore, the bullies were asked to come up with suggestions on how to improve the situation for the victim. The adults were requested not to place any blame on the bullies.

### Measure

The 11-item questionnaire delivered by the KiVa team members to the bullies opened with the following instructions: *Now you will be asked a few questions, which relate to your schoolmate’s problematic situation that has just been discussed. The answers will be sent to researchers who are interested in the thoughts and experiences of children of your age. When you have answered, put the paper in the envelope given to you and seal it. This way, no one in your school will know how you have answered.* The students were instructed to fill out the questionnaire in the same room where the individual discussion had been held and thereafter slip it into an envelope to ensure that the answers they provide would not be seen by the school personnel. At the end of the school year, all envelopes were delivered to the research team. For each of the 11 items, participants were asked to rate on a scale from 1 to 5 the extent to which they considered them to be true (1 = not true at all, 2 = not very true, 3 = cannot tell, 4 = quite true, 5 = completely true).

#### Empathy Arousal

The extent to which the bully perceived that the adult tried to raise his or her empathy for the victim was captured by the following four items: (a) *In the recent discussion, we talked especially about how bad my schoolmate is feeling*; (b) *the adult tried to make me understand how bad my schoolmate is feeling*; (c) *the adult did not blame me, but wanted me to help the schoolmate who is having a difficult time*; and (d) *the discussion helped me understand the difficult situation my schoolmate is in*. The Cronbach’s alpha for these four items was 0.76.

#### Condemning of the Behavior and Blaming of the Bully

A distinction was made between condemning of the bullying behavior and blaming of the bullying perpetrator. Condemning of the behavior was assessed by the following two items: (a) *During the discussion, it was clearly mentioned that I have behaved wrongly*, and (b) *during the discussion, the adult told me that he/she knew that I had been bullying my schoolmate and demanded that I stop*. These two items were highly correlated (*r* = .61; *p* < .001). One item assessed blaming of the bully: *The adult blamed me for things that have happened.* The correlations between this item and the two items assessing condemning of the behavior were 0.41 and 0.45 (*p*s < .001), respectively.

#### Self-Efficacy for Behavioral Change

The extent to which bullies felt capable of changing their conduct was assessed by the following three items: (a) *I believe I can help my schoolmate who is going through difficult times*; (b) *I feel that I now have means with which I can help my schoolmate who is having difficult times*; and (c) *I am good at comforting my mates*. The Cronbach’s alpha for these three items was .74.

#### Intention to Change Behavior

Bullies’ intention to change their behavior was measured by one item: *I believe that the discussion will affect my own behavior in the future.* This was the dependent variable of the study’s analyses.

## Results

### Analysis Strategy

The means, standard deviations, and correlations for all study variables are presented in Table 1. In order to examine whether and how perceived empathy arousal, condemning of the behavior, and blaming of the perpetrator relate to bullies’ intention to stop, we conducted a first regression analysis (model 1) with these three strategies as predictors and intention to stop as the dependent variable. In order to obtain the effects of each strategy regardless of the main approach adults had been instructed to use, we included type of approach as a covariate. We also controlled for school level (primary vs secondary) and feelings of self-efficacy for change. In order to test if the effects of empathy arousal depended on the extent to which bullies felt blamed and the extent to which they perceived the adult condemned their behavior, we conducted a second regression analysis (model 2). This model included the same predictors as model 1, as well as two interactions: Between empathy arousal and condemning of the behavior and between empathy arousal and blaming the child. In both models, all continuous predictors were mean centered. Results are presented in Table 2.

**Table 1:** Correlations among main study variables and means (and standard deviations) for the whole sample, by type of approach and by school level

	Correlations					Type of approach			School level		
	1	2	3	4	Overall	Confronting	Non-conf.	*t*	Primary	Secondary	*t*
1. Intent. change	–				4.12 (1.02)	4.22 (1.05)	4.05 (0.99)	1.447	4.10 (1.05)	4.14 (0.98)	0.412
2. Empathy arousal	0.51	–			4.08 (0.83)	3.96 (0.99)	4.16 (0.71)	2.029*	4.21 (0.79)	3.92 (0.86)	3.199**
3. Condemning	0.41	0.29	–		3.68 (1.24)	4.14 (1.03)	3.38 (1.27)	5.958***	3.77 (1.20)	3.58 (1.28)	1.413
4. Blaming child	0.09_a_	−0.01_a_	0.48	–	2.71 (1.43)	3.12 (1.48)	2.45 (1.34)	4.289***	2.64 (1.45)	2.80 (1.41)	1.017
5. Self-efficacy	0.48	0.57	0.26	0.01_a_	3.70 (0.78)	3.65 (0.83)	3.74 (0.75)	1.004	3.79 (0.81)	3.60 (0.73)	2.247*

*Note*. All correlations are significant at <.001, except for those with the subscript a, which are not significant

*Intent. change.* intention to change behavior, Condemning condemning of the behavior, *Non-confront.* non-confronting

*p < .05, **p < .01, ***p < .001

**Table 2:** Regression analyses predicting bullies’ intentions to change their behavior

	Model 1: main effects			Model 2: interactive effects		
	B	SE	*β*	*B*	SE	*β*
Intercept	40.058***			40.105***		
Type of approach	−0.093	0.098	−0.044	−0.112	0.097	−0.054
School level	0.255**	0.091	0.124	0.233*	0.091	0.114
Empathy arousal	0.392***	0.068	0.320	0.354***	0.069	0.289
Condemning behavior	0.226***	0.045	0.275	0.227***	0.045	0.275
Blaming child	−0.039	0.036	−0.055	−0.041	0.036	−0.058
Self-efficacy	0.312***	0.069	0.239	0.291***	0.070	0.223
Empathy arousal × condemning				−0.086*	0.040	−0.112
Empathy arousal × blaming child				−0.007	0.037	−0.010

The coding for type of approach was as follows: confronting 0 and non-confronting 1. The coding for school level was as follows: primary school 0 and secondary school 1. Interactions between grade level and each of the four predictors of interest (empathy arousal, condemning behavior, blaming child, and self-efficacy) were tested and were not significant. The interaction between empathy arousal and condemning behavior remains significant when these interactions are included in the model

*p < .05, **p < .01, ***p < .001

### Multiple Regression Analyses

#### Main Effects

Model 1 included main effects only and explained 39 % of the variance in intention to change behavior (adjusted *R* ^2^ = .38). There was no significant effect of the type of approach used on bullies’ intention to change their behavior, *p* = .343. There was a significant effect of school level: Children reported higher intentions to change their behavior in secondary school compared to primary school, *p* = .005. With regard to the strategies that bullies perceived the adults had used during the discussion, there was a positive effect of empathy arousal: The more bullies felt that teachers had tried to raise their empathy for the victim, the higher their reported intention to change, *p* < .001. Perceived condemning of the behavior also positively predicted intention to change, *p* < .001, but not perceived blaming of the bully, *p* = .276. Higher levels of self-efficacy were associated with a higher reported intention to change, *p* < .001.

#### Interaction Effects

**Fig. 2:**
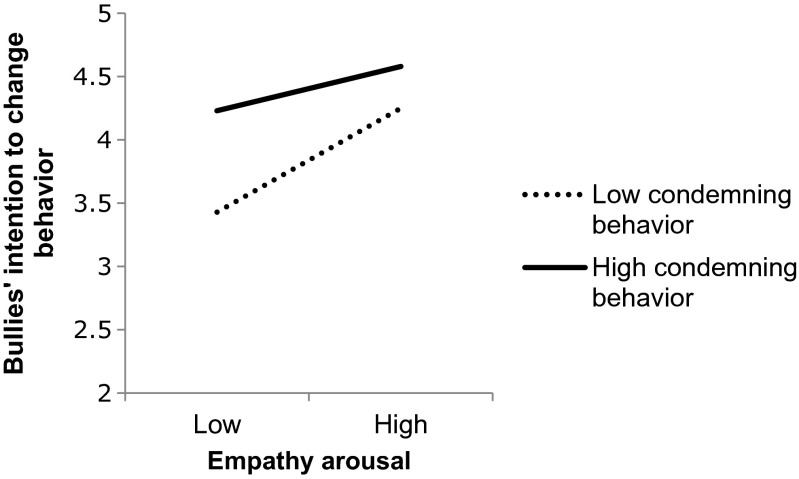
Moderating effects of condemning bullying behavior on the association between empathy arousal and bullies’ intention to change their behavior

Model 2 included the interaction between empathy arousal and condemning of bullying and the interaction between empathy arousal and blaming the child. It explained 40 % of the variance in the outcome (adjusted *R* ^2^ = .39). The only significant interaction was between empathy arousal and condemning of the behavior, *B* = −0.086, SE = 0.040, and β = −0.112; *p* = .031. First, the interaction was probed by calculating the slopes for the effects of empathy arousal on intention for behavioral change at high (M+1 SD) and low (M-1 SD) levels of the moderator (Aiken and West 1991). A graphical representation is shown in Fig. 2.

In order to examine the effects of the focal predictor at each value of the moderator and determine regions of significance, we used the SPSS macro MODPROBE developed by Hayes and Matthes (2009). Results showed that there were no region of significance for empathy arousal within the range of all possible values of condemning the behavior: The effects of empathy arousal are significant (and positive) at the lowest value of condemning the behavior, *B* = 0.586, SE = 0.114, *p* < .001, and at the highest value of condemning the behavior, *B* = 0.241, SE = 0.095, *p* = .012. This suggests that the effects of empathy arousal on intention to change are stronger when condemning the behavior is low, but remain significantly positive when condemning the behavior is high. We also examined the effects of condemning the behavior at each possible value of empathy arousal. Results also showed an absence of region of significance: The effects of condemning the behavior are significant (and positive) at the lowest value of empathy arousal, *B* = 0.493, SE = 0.132, *p* < .001, and at the highest value of empathy arousal, *B* = 0.147, SE = 0.057, *p* = .011. This suggests that the effects of condemning the behavior are stronger when empathy arousal is low but remain significantly positive when empathy arousal is high. Furthermore, bullies’ intention to change is the lowest (3.047) when both empathy arousal and condemning of the behavior are at their lowest level and the highest (4.642) when both empathy arousal and condemning of the behavior are at their highest level.

## Discussion

Extant literature on the optimal ways to handle cases of school bullying is relatively limited. The scientific debate on the topic has mainly opposed approaches that emphasize arousal of empathy and absence of blame to confrontational approaches that emphasize condemning of the behavior and blaming of the perpetrator. However, there is little empirical evidence regarding (a) the differential effects of condemning the bullying and blaming the bully, (b) the possible interactive effects of specific strategies (e.g., are the effects of raising empathy lower when bullies feel blamed?), and (c) the association between bullies’ own perception of these discussions and their intention to change their behavior. This study examined bullies’ perceptions of how they were treated by a school staff member during discussions that took place after a bullying incident came to their attention. Our first objective was to test how their perceptions of the extent to which the teacher aroused their empathy for the victim, condemned their behavior, and blamed them, affected their intention to change their behavior. Our second objective was to test if attempts at arousing empathy would be less effective when bullies also felt blamed by the teachers and/or felt that their behavior was condemned.

### Effects of Empathy Arousal, Condemning of Bullying and Blaming of the Bully

Both empathy arousal and condemning of the bullying positively predicted bullies’ intention to stop. Feeling blamed, however, had no significant effect. No evidence was found to indicate that the effectiveness of empathy arousal would be lower when bullies felt blamed by the adult. Feeling blamed neither decreased nor increased the effects of empathy arousal. Nevertheless, the effects of empathy arousal on bullies’ intention to change did vary depending on the extent to which bullies perceived that their behavior was condemned (and vice versa). When perceived empathy arousal was low, bullies reported a higher intention to change if their behavior was highly condemned rather than not condemned. If their behavior was not condemned, their intention to change was higher if attempts had been made to raise their empathy. Importantly, bullies’ intention to change was the highest when both empathy arousal and condemning of bullying was high.

Three main conclusions can be drawn from these findings. First, it is important to distinguish between condemning the behavior and blaming the child. While condemning the behavior yields desirable effects, blaming the child does not. Second, it is advantageous that interventions directed at bullies combine efforts at raising bullies’ empathy for the victim with a clear condemnation of their behavior. Finally, the finding that the effects of empathy arousal are stronger (i.e., steeper) when blaming the behavior is low (and vice-versa) implies that adults in school should make sure that they do at least one of these two things when tackling bullying. If they do not attempt to raise the empathy of the child, it is particularly important that they condemn the behavior; if they do not condemn the behavior, they should put special effort into raising the perpetrator’s empathy.

### Limitations and Future Research

Although the present study addresses important gaps in the anti-bullying literature, it has several limitations. We measured bullies’ perceptions of how they were treated by the adult during intervention discussions, but whether these perceptions accurately reflect what the adults said remains unknown. For instance, the extent to which bullies felt blamed may have been influenced by their own processing of social information, such as a hostile attribution bias. For the current findings to be translated into recommendations for practice, it would be important to determine the level of objectivity of bullies’ perceptions and how it relates to their intentions and behaviors. Future studies should consider the systematic recording of the discussions to provide a validity check of these perceptions.

In order to prevent social desirability bias, the questionnaire administered to the bullying students was anonymous. For this reason, it was not possible to examine whether individual characteristics of the respondents were associated with their intention to stop their behavior. Future research should determine for instance if the effect of empathy arousal on decreases in bullying depends on the initial level of empathy of the bully. In some cases, young bullies may already have a good understanding of the suffering that the victim is experiencing; “ringleader” bullies have been found to outscore “follower” bullies in emotions’ understanding (Sutton et al. 1999). Therefore, we might expect empathy arousal to be more effective among students who assist in bullying rather than initiate it. Similarly, the KiVa program as a whole was found to be less effective among highly popular bullies (Garandeau et al. 2014a). Bullies who are socially rewarded with high peer status might be less responsive to adults’ open disapproval of their behavior.

A third limitation of the study is that questionnaires were received from only 36 % of the participating schools. As the focus of this study was in the association between specific components of two bully-targeted intervention approaches and bullies’ intention to stop, this rather low percentage should not significantly affect the validity of our findings. However, it does raise the questions of whether the schools who did not return the questionnaires still held the discussions, whether the personnel in those schools were less receptive to bullying behaviors or whether there were fewer bullying incidents occurring in those schools. Furthermore, for primary school grades, the number of questionnaires received from schools using the non-confronting approach was twice as high as the number of questionnaires received from schools using the confronting approach. The reason for this is unclear; one explanation is that the use of the non-confronting approach requires less strict evidence of what happened compared to the confronting approach. Therefore, this approach may have been applied to cases that did not strictly fulfill the criteria of bullying.

With regard to the instrument itself, three limitations should be noted: First, both blaming of the child and intention to change were measured with a single item. Their reliability is therefore unknown. Second, the distinction between condemning the behavior and blaming the child may not have been perfectly operationalized: The difference between “The adult knew I have been bullying” and “The adult blamed me” is subtle. The fact that the two items are differentially correlated to the outcome measure indicates that they were distinguished by the bullies. However, blaming of the child implies condemning of the behavior, which makes their operationalization difficult. Third, no distinction was made between affective and cognitive empathy in the measurement of empathy arousal. As these two components of empathy have been found to be differentially associated with bullying behaviors (Caravita et al. 2009; Jolliffe and Farrington 2011), it seems that this distinction should be taken into account in interventions designed at increasing it. On the other hand, van Baardewijk et al. (2009) argue that the differentiation between the cognitive and affective elements of empathy may not be highly relevant to the extent that these elements are not independent of each other. On the contrary, cognitions and affective responding combine to influence behavior.

Finally, the operationalization of the outcome measure as self-reported intention to change bullying behavior instead of actual change in behavior is an important limitation. Despite the anonymity of the questionnaire, the possibility that participants responded in a way that they felt was expected of them, cannot be ruled out. This may partly explain the high mean for the reported intention to change. Moreover, bullies who felt blamed for who they are may be less willing than bullies whose behavior was condemned to report that they will change, but we cannot be certain that their bullying decreased less. Although it is likely that the reported intention to behave a certain way is positively associated with actual behavior (Sheeran 2002; Webb and Sheeran 2006), future studies should examine the effects of these various intervention components on peer-reported bullying behavior.

The improvement of anti-bullying interventions calls for a better understanding of the processes underlying their effectiveness. The current study sheds a light on these mechanisms by showing that bullies’ intention to change their behavior following a discussion with a school personnel member is higher when they perceive that the adult conducting the intervention attempted to raise their empathy for the victim and condemned their behavior. Current findings demonstrate that the debate on bully-targeted interventions, which focused on the no-blame versus disciplinary strategy dichotomy, may be misguided. Combining attempts at raising empathy and clear reprobation of the bullying behavior without blaming the perpetrator himself may result in the largest decrease in bullying. More research is needed on possible moderators of the effects of these specific intervention components, as well as on their long-term effects on actual behavior.
